# Stable Cu Isotope Ratios Show Changes in Cu Uptake and Transport Mechanisms in *Vitis vinifera* Due to High Cu Exposure

**DOI:** 10.3389/fpls.2021.755944

**Published:** 2022-01-12

**Authors:** Simon Blotevogel, Priscia Oliva, Laurence Denaix, Stéphane Audry, Jerome Viers, Eva Schreck

**Affiliations:** ^1^Géosciences Environnement Toulouse (GET), Université Paul-Sabatier Toulouse III, CNRS, IRD, Toulouse, France; ^2^Interactions Sol Plante Atmosphère (ISPA), Institut National de Recherche Pour l’Agriculture, l’Alimentation et l’Environnement (INRAE), Bordeaux Sciences Agro, Villenave d’Ornon, France

**Keywords:** *Vitis vinifera*, copper, metal stress response, soil solution (pore water), bioavailability, translocation, grapevine, humic acid

## Abstract

Even though copper (Cu) is an essential plant nutrient, it can become toxic under certain conditions. Toxic effects do not only depend on soil Cu content, but also on environmental and physiological factors, that are not well understood. In this study, the mechanisms of Cu bioavailability and the homeostasis of *Vitis vinifera* L. cv. Tannat were investigated under controlled conditions, using stable Cu isotope analysis. We measured Cu concentrations and δ^65^Cu isotope ratios in soils, soil solutions, roots, and leaves of grapevine plants grown on six different vineyard soils, in a 16-week greenhouse experiment. The mobility of Cu in the soil solutions was controlled by the solubility of soil organic matter. No direct relationship between Cu contents in soils or soil solutions and Cu contents in roots could be established, indicating a partly homeostatic control of Cu uptake. Isotope fractionation between soil solutions and roots shifted from light to heavy with increasing Cu exposure, in line with a shift from active to passive uptake. Passive uptake appears to exceed active uptake for soil solution concentrations higher than 270 μg L^–1^. Isotope fractionation between roots and leaves was increasingly negative with increasing root Cu contents, even though the leaf Cu contents did not differ significantly. Our results suggest that Cu isotope analysis is a sensitive tool to monitor differences in Cu uptake and translocation pathways even before differences in tissue contents can be observed.

## Introduction

Grapevine plants are commonly sprayed with copper (Cu) based fungicides, including the Bordeaux mixture (CuSO_4_, combined with lime), Cu-oxides (Cu_2_O, CuO), and Cu-hydroxide (Babcsanyi, 2015; Blotevogel et al., 2018). These fungicides have been used for more than 150 years and are still the only permitted treatment against downy mildew in organic viticulture. The long-term treatment of vineyards with Cu-based fungicides led to increased Cu contents, especially in topsoils (Flores-VéLez et al., 1996; Brun et al., 2001; Chaignon et al., 2003; Pietrzak and McPhail, 2004; Duplay et al., 2014). Even if copper is an essential nutrient for plants, it can become toxic if it is available in excess (Marschner and Marschner, 2012). The grapevine plant was long believed to be tolerant to high Cu exposure but reports of negative side effects are increasing and some vineyards experience problems during replantation (Toselli et al., 2009; Anatole-Monnier, 2014; Martins et al., 2014; Miotto et al., 2014; Ambrosini et al., 2015; Brunetto et al., 2016). The mobility and phytoavailability of Cu in soils and its uptake by plants are complex and not only depending on soil or plant properties but also on their interaction (Yin et al., 2002; Kabata-Pendias, 2004; Ma et al., 2006; Wang and Staunton, 2006; Bravin et al., 2009a,^2012^).

In soils, Cu shows a strong chemical affinity for the particulate phase (e.g., oxyhydroxides and organic matter) (Chaignon et al., 2003; Bradl, 2004; Pietrzak and McPhail, 2004; Strawn and Baker, 2008, 2009; Sayen et al., 2009; Sayen and Guillon, 2010; Abgottspon et al., 2015). The Cu content in soil solutions is generally low compared with the bulk soil content and is mainly controlled by the presence of soluble organic ligands and pH (Lexmond, 1980; Yin et al., 2002; Ashworth and Alloway, 2004). In the direct vicinity of roots – the rhizosphere, physico-chemical conditions as pH, Eh, and presences of organic ligands can be modified by plants to meet their mineral nutrition needs (Hinsinger et al., 2003; Kraemer, 2004; Bravin et al., 2009a,^2012^). On one hand, this can be used to limit toxicity, durum wheat for example drastically reduced the Cu bioavailability in contaminated soils by rhizosphere alkalization (Hinsinger et al., 2003; Bravin et al., 2009a,^2012^). On the other hand, the solubility of scarcely available nutrients such as iron (Fe) can be increased by exudation of reducing agents and phytosiderophores (Kraemer, 2004; Schenkeveld and Kraemer, 2018). Note that both strategies are not ion-specific and will affect other elements as well (Hinsinger et al., 2003; Bravin et al., 2009a; Schenkeveld and Kraemer, 2018).

**FIGURE 1:**
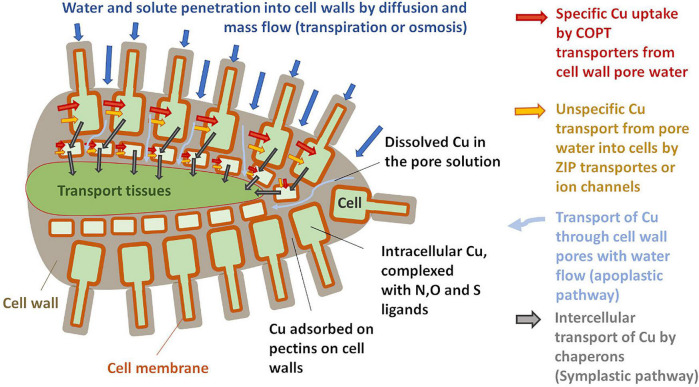
Schematic sketch of copper (Cu) uptake and transport in roots (drawn after Printz et al., 2016; Kim et al., 2018).

The plant uptake of metal from the soil solution depends on its concentration and speciation in the rhizosphere, on the plant species and nutrition status, and the availability of other nutrients (Yin et al., 2002; Bravin et al., 2009a,^2012^; Toselli et al., 2009). Driven by diffusion or mass flow, ions and small complexes (<5 nm) from the soil solution enter the pores of the cell walls and penetrate far into the plant roots (Figure 1) *via* the so-called apoplastic pathway (Marschner and Marschner, 2012). Metal ions can be adsorbed to pectins in the cell wall and it was reported for high Cu exposures that large fractions of root Cu are sequestered by cell wall adsorption (Shi et al., 2011; Li et al., 2016; Wan et al., 2019; Cui et al., 2020). Nevertheless, the apoplast is a dynamic compartment and it was reported that plants can adjust the number and type of metal-binding pectins as a response to metal stress (Sattelmacher, 2001; Castaldi et al., 2010; Colzi et al., 2012; Meychik et al., 2016). Some 10–20% of the apoplastic Cu was reported to be in the reduced form of Cu(I) (Cui et al., 2020). Note that *in situ* measurements of compartmentalization and speciation in roots were usually carried out on plants that received high Cu doses to overcome experimental detection limits.

From the pore solution of the apoplast, the uptake of Cu into the root cells can be mediated by several transporters (Figure 1). Most important under low Cu availability are high-affinity transporters of the COPT/Ctr protein family (Martins et al., 2014; Printz et al., 2016). Those highly specific Cu transporters require the reduction of Cu into Cu(I) by ferric reductase oxidase (Bernal et al., 2012; Jouvin et al., 2012; Ryan et al., 2013). This is backed by the XANES analysis that shows large proportions of Cu(I) in plant roots and implies that the homeostasis of Cu is closely linked to that of Fe (Bernal et al., 2012; Ryan et al., 2013; Printz et al., 2016; Cui et al., 2020). Besides this high-affinity transport system, Cu(II) is likely also taken up through ZIP transporters and ion channels that can carry different divalent cations (Printz et al., 2016). At very high Cu exposure levels, plants might be able to efflux Cu from the root cells (Burkhead et al., 2009; Printz et al., 2016).

The Cu can then be transported from cell to cell or within the cells by Cu-specific chaperon proteins (Mira et al., 2001; Wintz and Vulpe, 2002; Printz et al., 2016). In plant fluids, Cu is likely to be present only in organometallic complexes that provide both solubility and shielding during long-distance transport (Alvarez-Fernandez et al., 2014 and references therein). Such complexes prevent toxicity as they are less reactive than free metal ions (Burkhead et al., 2009; Guigues et al., 2016). To reach the xylem that transports Cu up into the leaves, Cu coming from both symplastic and apoplastic pathways needs to be transported across the Casparian strip, a diffusion barrier for solutes (Printz et al., 2016; Kim et al., 2018). From there Cu is translocated towards the leaves *via* the xylem sap.

Meanwhile, isotope ratios of Cu have been used to trace Cu dynamics in soils (Bigalke et al., 2010b; Fekiacova et al., 2015; Babcsányi et al., 2016; Vance et al., 2016; Kusonwiriyawong et al., 2017; Blotevogel et al., 2018) and plants (Weinstein et al., 2011; Jouvin et al., 2012; Ryan et al., 2013; Li et al., 2016; Blotevogel et al., 2019). While soil processes regulating Cu mobility and speciation, as complexation with organic ligands, redox reactions, and mineral dissolution, induce limited Cu-isotopic fractionation (<1‰), Cu uptake and translocation in plants have been observed to cause large fractionations up to −1.43‰ (Bigalke et al., 2010a; Weinstein et al., 2011; Jouvin et al., 2012; Mathur et al., 2012; Ryan et al., 2013; Babcsányi et al., 2016; Li et al., 2016; Blotevogel et al., 2018, 2019). Several studies investigated Cu isotope fractionation in plants under hydroponic conditions and contributed to the understanding of redox steps during uptake by consistently reporting light isotope uptake (Jouvin et al., 2012; Ryan et al., 2013). Both studies report different fractionation patterns for root to shoot transport, which are likely due to differences in speciation of supplied Cu (ionic versus complexed) or root washing and desorption protocols (Jouvin et al., 2012; Ryan et al., 2013). Hydroponic conditions do not perfectly mimic natural systems especially they dilute root exudates and modify nutrient balance and supply mechanisms as well as Cu speciation in solution. In former studies, field-grown plants showed stronger isotope fractionation (up to −2‰) than were observed in hydroponic studies (Weinstein et al., 2011; Blotevogel et al., 2018, 2019).

Thus, to better understand the mechanisms of Cu-availability in soils, its uptake and translocation into the plant under increasing Cu-concentrations in vinyard soils, we performed a greenhouse experiment with pot-grown grapevine plants (i.e., *Vitis vinifera* L. cv. Tannat). Cu-concentrations and δ^65^Cu isotope ratios were measured in the soils, soil solution, roots, and leaves of plants grown in six different soils presenting variable Cu pesticide background and pedological characteristics. Soil solutions were sampled every two weeks and grapevine plants were destructively harvested for analysis after 16 weeks. We thus aimed to show that the mechanisms of Cu mobility and homeostasis can be efficiently monitored by Cu stable isotope fractionation.

## Materials and Methods

### Soil Description

The six selected surface soils (0–20 cm) were sampled from three winegrowing areas in France and one in Italy, presenting different soil types, physico-chemical properties, and in particular different Cu treatment histories (Table 1). Among the three soils coming from the “Bordeaux” area, two of them (HBN, CO) have been used for viticulture and received Cu fungicide application for over 100 years. Replantation of grapevine was reported to be problematic for these soils, likely due to Cu toxicity to young plants (details are published in Anatole-Monier in 2014). The third soil from the “Bordeaux” area (OB) was a forest soil that was recently converted to conventional viticulture including the use of Cu-based fungicide (conversion 4 years before sampling). The two soils from the “Soave” region (CI, VI) in northern Italy have received Cu fungicide applications for about a century but there was no evidence of toxicity for grapevines. The last selected soil (STM) is a vineyard soil from the Saint Mont region (STM), which did not receive any Cu treatment.

**TABLE 1:** Pedological, mineralogical and physico-chemical properties of the studied vineyard soils.

Soil ID	Area	Type of viticulture	Soil type	Main mineral phases	pH	CEC	SIC	SOC
						cmol kg^–1^	% wt	% wt
CO	Bordeaux, France	Conventional	Fluvisol	Qtz.	7.2	5.2	<0.1	0.6
HBN	Bordeaux, France	Conventional	Fluvisol	Qtz.	7.4	7.7	0.1	1.3
OB	Bordeaux, France	Conventional	Fluvisol	Qtz.	7.6	3.8	<0.1	0.5
CI	Soave, Italy	Organic	Calcaric Cambisol	Calc., Fels., Smec.	7.8	49.9	5.1	2.1
VI	Soave, Italy	Organic	Vertic Cambisol	Fels., Smec., Qtz.	7.7	59.8	0.4	2.5
STM	Saint Mont, France	Conventional (no Cu use)	Ferric Gleysol	Qtz.	6.6	5.5	<0.1	<0.1

*Soil types are given according to the world reference base (WRB) (FAO, 2014), CEC, SIC, and SOC are abbreviations for cation exchange capacity, soil inorganic carbon, and soil organic carbon, respectively. Qtz. = Quartz, Calc. = Calcite, Smec. = Smectite, Fels. = Feldspar.*

The soil samples were air-dried and sieved to 2 mm. The soil pH was measured on 1 g in 5 mL ultrapure water (18.2 MΩ) following the ISO 11464 protocol. The cation exchange capacity (CEC) was determined using cobalt hexamine. Therefore, 1 g of soil was shaken in 20 mL of a 0.017 mol L^–1^ cobalthexamine solution for 1 h, solutions were subsequently centrifuged and the supernatant filtered at 0.22 μm. Then, the cobalthexamine loss from the solution was determined by absorbance loss at 475 nm with a Varian Cary 50 spectrophotometer, Varian, Palo Alto, CA, United States. The total soil organic carbon (SOC) and total soil inorganic carbon (SIC) were calculated after subsequent measures of raw and calcined samples on a EMIA – 320 V CS automate, by Horiba Kyoto, Japan.

### Experimental Design

**FIGURE 2:**
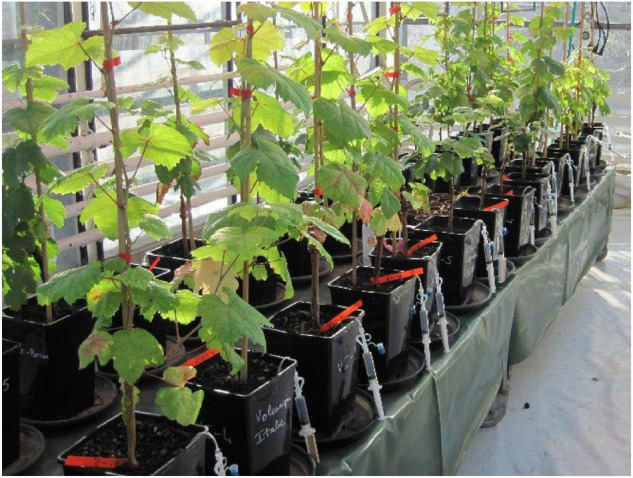
Picture of the grapevine plants and the soil solution sampling device, just before harvest. Plant height was about 1.2 m.

Composite samples (100 kg) of the first 20 cm of each soil were taken during winter and spring 2016 before the first annual fungicide treatment. The soils were root-picked, dried at room temperature, and sieved with a 2-mm mesh before potting. Soil samples were then filled into 5 L PVC (polyvinyl chloride) pots and one young vine plant (*Vitis vinifera* L. cv. Tannat grafted on rootstock *V. riparia* × *V. rupestris* cv. 101.14) was planted into each pot. Five replication pots were prepared for each soil modality and a microporous cup (RHIZON^®^ MOM 10 cm, Rhizosphere Research Products, pore size ∼0.1 μm) was inserted into each pot for sampling soil solution (Figure 2). After planting, pots were saturated with water and placed on heated ground until bud break (5d). Then, pots were placed in a greenhouse at the ISPA laboratory (INRAE Institute, Bordeaux, France) under artificial lighting mimicking 12 h of daily sunshine (Figure 2). The plants were regularly watered using demineralized water to maintain soil moisture at 80% of the water holding capacity of each soil.

### Sampling of Soil Solution and Plant Tissues

Soil solution sampling began one week after potting and from then on samples were taken every two weeks. Samples were taken by applying under pressure to the suction cups for 10 min, thereby extracting between 0.5 and 10 mL of solution. The first two samples (week 0 and week 2) were not included in the analysis, to let the system equilibrate. From week 4 to 16, the soil solution samples were collected and the total organic carbon (TOC) and total inorganic carbon (TIC), as well as pH, were measured promptly after sampling. Leftover solutions were acidified with ultrapure nitric acid to 2% (v/v) for later analyses of Cu concentration and isotope ratio.

Initially, five plants were grown per modality to be sure to have at least three healthy plants, as some soils were reported to be problematic for replantation. However, only three healthy plants per soil modality were destructively harvested after 16 weeks (see Figure 2 for plants just before harvest). Five healthy fully unfolded leaves of every branch counting from the top were cut using a ceramic knife and put into plastic sample bags for Cu content and isotope ratio measurements.

Roots were extracted from soil and washed under flowing demineralized water. When no more soil particles were visible, the roots were cut from the trunk and put into sample bags. Root and leaf samples were then washed three times using demineralized water and twice using ultrapure water (18.2 MΩ). All samples were frozen at −80°C and freeze-dried. Once dried, root and leaf samples were ground to powder using a planetary mill with Zr-containers and balls.

### Determination of Cu Concentrations in Soil, Soil Solution, and Plant Samples

Sample digestion was carried out in the ISO 7 cleanroom laboratories of the GET Toulouse and the LEGOS Toulouse. A 100 mg sample of each ground soil was digested in a MARS 5 microwave oven (by CEM, Matthews, NC, United States) using ultrapure acids (9 mL HNO_3_: 2 mL HCl: 3 mL HF), the solution was then evaporated and the samples were dissolved in double subboiled HNO_3_ for analysis. Details of the digestion protocol were published elsewhere (Blotevogel et al., 2018). The plant samples were digested on hotplates in three steps. For each plant sample, 200 mg of powder were weighed into Savillex Teflon vessels. Then, 1 mL of ultrapure hydrogen (H_2_O_2_) was added and left to react for 2 h at room temperature. Subsequently, 5 mL of double subboiled nitric acid (HNO_3)_ were added in 1 mL steps to each vessel and left to react overnight. Successively, the vessels were heated to 120°C for at least 4 h and evaporated to dryness at 90°C. Once dried, 4 mL of double subboiled HCl and 2 mL of double subboiled HNO_3_ were added along with 1 mL of suprapure HF. Finally, vessels were heated to 120°C for at least 4 h and evaporated to dryness at 90°C. A final digestion step was performed using 5 mL HNO_3_, the samples were again heated to 120°C for at least 4 h and evaporated to dryness at 90°C.

All soil solution samples with sufficient volume (more than 100 μL) were analyzed for their Cu concentration. During the whole experiment, 1 replicate for OB, 2 for CO, 5 for CI and VI, 6 for HBN, and 9 replicates for STM could not be analyzed for their Cu content, either due to handling errors or too low sample volume (see Supplementary Information for details).

In soil soultions and digested soil and plant samples, total Cu concentrations were measured by inductively coupled plasmamass-spectrometry (ICP-MS) (7500ce, Agilent Technologies – Santa Clara, CA, United States) and inductively coupled plasma – optical emission spectrometry (ICP-OES) (Ultima Expert, Horiba Jobin Yvon, Kyoto, Japan) at the GET laboratory. The analytical accuracy and precision of measurements were ensured by measuring replicates of the SLRS-5 river water standard (*n* = 6). Relative standard deviations (RSD%) were <5% and Cu-recovery 100 ± 5%. The accuracy and precision of the whole sample preparation procedure were checked by determining Cu concentration in BCR-2 basalt standard [Cu recovery of 96 ± 10% (*n* = 3)] and SRM-1515 apple leaf standard [Cu recovery of 92 ± 5% (*n* = 4)].

### Isotope Analyses in Soil, Soil Solution, and Plant Samples

Cu isotope measurement by multicollector-inductively coupled plasma mass spectrometer (MC-ICP-MS) (Neptune, by Thermo Fisher, Bremen, Germany) required at least 500 ng of Cu. When the amount of Cu was insufficient in a single soil solution sample, we chose to pool all replicates from a given sample week (e.g., STM and VI modalities and the late samples of OB). This allowed us to have at least one soil solution Cu isotope measurement per modality at each sample time.

Purification of digested samples was carried out under ISO1 laminar flow hoods using anionic AG MP-1 resin (Bio-Rad PolyPrep chromatographic columns – Hercules, CA, United States) (Maréchal et al., 1999). Volumes of resin and solution were adapted for the different matrices to ensure quantitative elution, as the separation procedure fractionates Cu isotopes up to ≈ 19‰, between the first and the last mL of Cu elution (Maréchal and Albarède, 2002). Soil samples were purified according to the protocol described in Blotevogel et al. (2018) and displayed in the supporting information (Supplementary Information Table 1). Soil solution aliquots were digested in three steps as described above for plant samples. For purification of soil solution, root, and leaf samples, the same Bio-Rad Poly Prep chromatographic columns were filled with 2 mL of AG MP-1 resin and conditioned according to Maréchal et al. (1999). After sample loading using 1 mL of a 7 M HCl solution containing 0.01% of H_2_O_2_, the matrix was eluted by passing 9 mL of the same 7M HCl, 0.01% H_2_O_2_ solution. Cu was then eluted using 24 mL of the same solution (Supplementary Information Table 1). This same purification procedure was carried out twice for soil solution, root, and leaf samples. Details on the method development and elution profiles can be found in Blotevogel (2017). Cu isotope ratios are expressed in ‰ relative to NIST 976 Cu standard (Eq. 1).


(1) δ65⁢C⁢u=((C65⁢u/C63⁢u)S⁢a⁢m⁢p⁢l⁢e(C65⁢u/C63⁢u)N⁢I⁢S⁢T⁢976-1)*1000

Recovery of all purified samples was checked to be 100 ± 5%. BCR-2 standards were used as precision and accuracy control of the whole procedure, the reference material was digested three times and four different purification runs were performed allowing 10 measurements. BCR-2 isotope ratios (0.26 ± 0.09‰) were slightly heavier than values from literature 0.20 ± 0.10‰ (Babcsányi et al., 2014) and 0.22 ± 0.05‰ (Bigalke et al., 2010a) but within the 2SD range of those results.

### Statistical Analyses

All data analysis was carried out using the R software^®^ in version 4.0.3. Plant biomass and Cu concentration data were analyzed using a one-way ANOVA. Except for Cu concentrations in roots ([Cu]_Roots_) and in soil solutions ([Cu]_Solution_) there was no departure from normality of residues within a 95% confidence interval using a Shapiro-Wilk test. To achieve normality, the [Cu]_Roots_ and [Cu]_Solution_ datasets were submitted to a logarithmic transformation and afterward satisfied the above-mentioned criteria. The one way ANOVAs were computed to determine if the soil type had a significant influence on the data set. Differences between means were then tested using Tukey’s HSD. Differences between groups are shown in compact letter display computed using Tukey’s HSD with *p* < 0.05. For soil solution samples, a one-way ANOVA was computed for each soil type to detect if there were significant differences in Cu concentration over time.

Isotope data were reported using the mean value of the three replicate samples and a 2 standard deviation (SD) error bar around this mean. Differences were considered as significant if 2SD intervals did not overlap. For isotope fractionation data the standard deviation of replicate measurements was propagated.

## Results

### Mobility of Cu Into the Soil Solution and Its Evolution Over Time

Copper (Cu) concentrations in bulk soils ([Cu]_Soil_) were between 3 and 251 mg kg^–1^ (Table 2). OB and STM had the lowest [Cu]_Soil_ (3 and 10 mg kg^–1^, respectively), whereas CO, CI, VI, and HBN soils had higher [Cu]_Soil_ of 115, 214, 229, and 251 mg kg^–1^, respectively.

**TABLE 2:** Mean Cu concentrations and isotopic ratios in the different compartments (soil, soil solution, roots, and leaves) of the different soil modalities (STM, VI, CI, OB, HBN, CO). For soils also SOC values are given and in soil solutions pH and TOC.

		Type	STM	VI	CI	OB	HBN	CO
SOC	wt.%	Soil	0.1	2.5	2.1	0.5	1.3	0.6
TOC	mg L^–1^	Solution	43	41	62	102	76	97
pH		Solution	6.4	7.9	7.7	8.0	7.8	7.9
Cu	mg kg^–1^	Soil	10	229	214	3	251	115
Mean Cu ± SD	μg L^–1^	Solution	19 ± 9 a	40 ± 15 b	100 ± 37 c	297 ± 247 d	999 ± 680 e	2,705 ± 1,215 f
Min-Max Cu	μg L^–1^	Solution	11−30	28−61	66−170	21−816	469−1,671	794−6,060
Mean Cu ± SD	mg kg^–1^	Roots	30 ± 8 a	81 ± 20 b	199 ± 27 c	25 ± 5 a	768 ± 114 d	579 ± 156 d
Min-Max Cu	mg kg^–1^	Roots	24–39	68–103	169–220	21–31	654–883	403–701
Mean Cu ± SD	mg kg^–^1	Leaves	5.1 ± 1.1 a	7.4 ± 2.1 ab	6.7 ± 1.1 ab	4.9 ± 2.2 ab	6.1 ± 1.2 ab	10.9 ± 3.1 b
Min-Max Cu	mg kg^–1^	Leaves	3.9–5.9	6.0–9.8	5.4–7.5	3.4–6.5	5.1–7.4	7.8–14.0
δ^65^Cu ± 2SD	‰	Soil	0.17 ± 0.11	0.33 ± 0.01	0.21 ± 0.04	0.27 ± 0.24	0.12 ± 0.07	0.02 ± 0.09
δ^65^Cu* ± 2SD	‰	Solution	0.61 ± 0.08	0.56 ± 0.11	0.55 ± 0.11	0.21 ± 0.14	0.05 ± 0.11	−0.02 ± 0.11
^65^Cu ± 2SD	‰	Roots	0.25 ± 0.04	0.30 ± 0.05	0.37 ± 0.03	0.26 ± 0.08	0.30 ± 0.20	0.24 ± 0.18
δ^65^Cu ± 2SD	‰	Leaves	0.20 ± 0.07	0.00 ± 0.16	−0.08 ± 0.30	0.24 ± 0.01	−0.20 ± 0.17	−0.14 ± 0.25
Δ^65^Cu* ± 2SD	‰	Solution*-Soil	0.44 ± 0.14	0.23 ± 0.11	0.34 ± 0.12	−0.06 ± 0.28	−0.07 ± 0.13	−0.04 ± 0.14
Δ^65^Cu* ± 2SD	‰	Root-Solution*	−0.36 ± 0.09	−0.26 ± 0.12	−0.18 ± 0.11	0.05 ± 0.16	0.25 ± 0.23	0.26 ± 0.21
Δ^65^Cu ± 2SD	‰	Leaves-Roots	−0.05 ± 0.08	−0.30 ± 0.17	−0.45 ± 0.30	−0.02 ± 0.08	−0.50 ± 0.26	−0.38 ± 0.31

Mean Cu and standard deviation (SD) values were calculated using all available Cu analyses (25 < n < 33 in soil solutions and n = 3 in plant tissues). The compact letter displays behind mean Cu values indicate significant differences between groups calculated using Tukey’s HSD with an alpha of 0.05. The 2SD values in isotope analysis correspond to a 2SD interval around the mean, calculated using isotope ratio analysis of all replicates (n = 3 for plant samples and n = 1, 2, 3, 3, 8, 12, for isotope analysis in STM, VI, CI, OB, HBN, and CO soil solutions, respectively, note that STM and VI solution were pooled over multiple replicates and time steps). For the soil samples, only one sample was analyzed in the beginning of the experiment and the 2SD interval corresponds to repeated isotope measurements including sample preparation. Δ^65^Cu values correspond to the Cu isotope fractionation between two compartments. δ^65^Cu* are mean Cu isotope composition for soil solutions calculated without samples from week 4.

**FIGURE 3:**
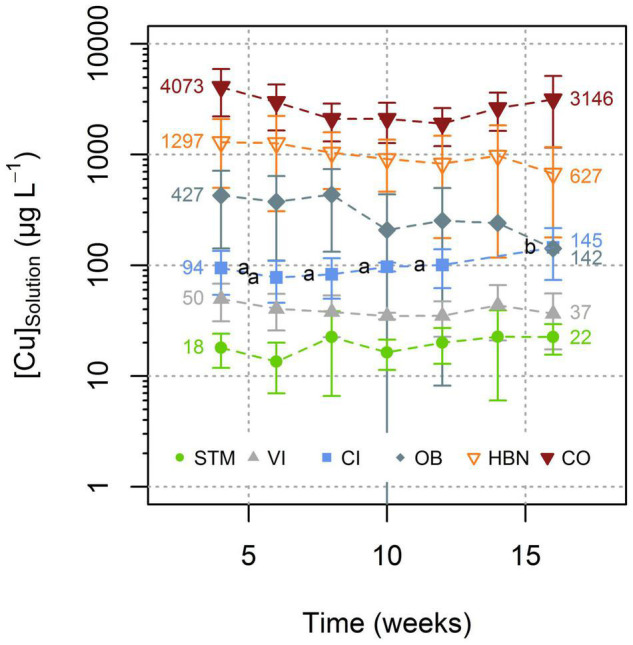
Evolution of Cu concentration in soil solutions with time. Reported results for each time point corresponding to the mean value of Cu concentrations in all replicates for each soil type (see Supplementary Information 1 for detailed values). Error bars correspond to the 2SD interval around the mean. Only for CI significant evolution over time was detected (Tukey’s HSD, α = 0.05). Groups are displayed as letters beside the respective points.

During the experiment, for each soil modality, the [Cu]_Solution_ remained in the same order of magnitude (Figure 3). Only for CI the one-way ANOVA showed a significant influence of the time on [Cu]_Solution_ (*p* < 0.0002) and Tukey’s HSD indicated that the solutions of week 16 had significantly (*p* < 0.02) higher Cu concentrations than solutions from all other time steps (Figure 3).

**FIGURE 4:**
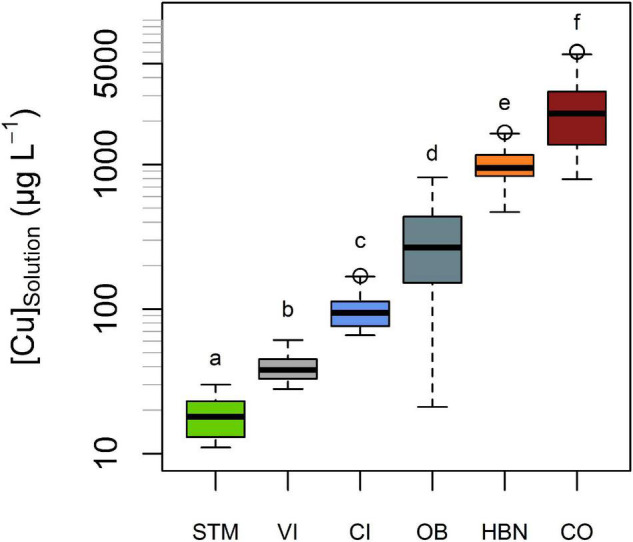
Cu concentrations (expressed in μg L^–1^) in solution denoted [Cu]_solution_, in the six different soils. All measurements of soil solution in each soil, including different points in times and replicates, are represented in this plot. The bold line represents the median value and the boxes include values between the 25th and 75th percentile, the y-axis is in log-scale. [Cu]_solution_ significantly (Tukey’s HSD, α = 0.05) differed from each soil to each soil. Groups are shown by letters above the boxes.

The lowest mean [Cu]_Solution_ was measured in STM soils (19 μg L^–1^), followed by CI and VI soils with mean [Cu]_Solution_ of 40 and 100 μg L^–1^, respectively (Figure 4). The highest [Cu]_Solution_ values were measured in soils from the “Bordeaux” area with 297, 999, and 2,705 μg L^–1^ (for OB, HBN, and CO, respectively). The differences between different modalities were much larger than variations over time within the same soil (Figure 4). A one-way ANOVA showed a significant influence of soil type on [Cu]_Solution_ (*p* < 10^–15^) and Tukey’s HSD indicated that mean [Cu]_Solution_ for each soil modality was significantly (*p* < 10^–5^) different from all other soil modalities (Figure 4).

**FIGURE 5:**
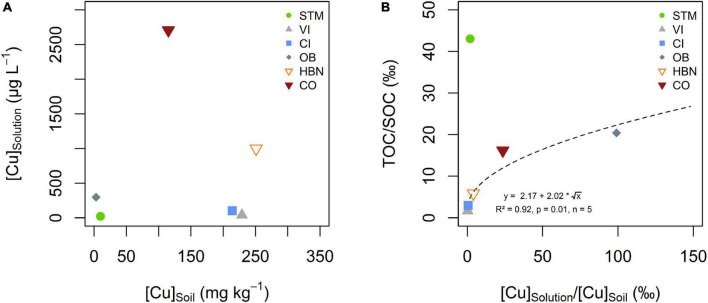
**(A)** Bulk soil Cu concentration ([Cu]_soil_) plotted against the arithmetic mean of soil solution Cu concentration ([Cu]_solution_). **(B)** Ratios of solution total organic carbon (TOC) over SOC plotted against mean solution Cu over soil Cu content for the different soil modalities. The dashed line represents the best fit of a square root function on the data excluding STM.

There was no direct correlation between [Cu]_Soil_ and [Cu]_Solution_ (Figure 5A), also between the TOC of the soil solution and [Cu]_Solution_ no direct correlation could be established, even when STM was excluded from the fit. Nevertheless, there was a positive relationship between mobile SOM (expressed as TOC/SOC) and mobile Cu (expressed [Cu]_Solution_/[Cu]_Soil_), when STM was excluded (Figure 5B, *R*^2^ = 0.92, *p* = 0.01, *n* = 5).

**FIGURE 6:**
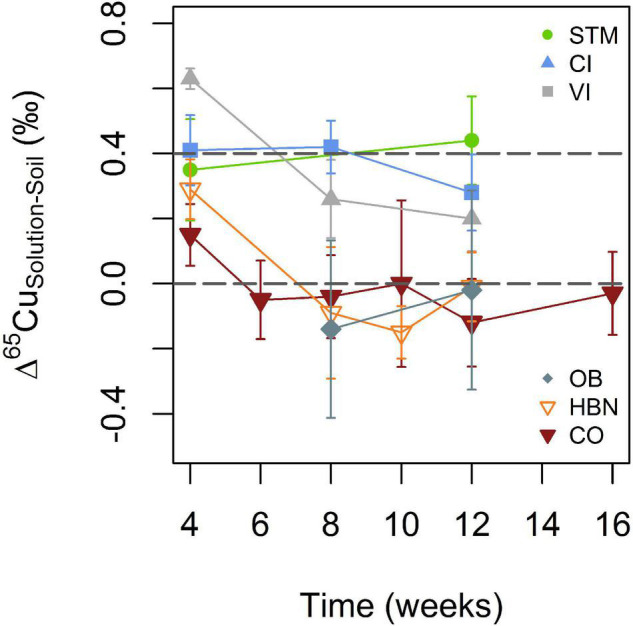
Evolution of Cu isotope fractionation between soil solutions and bulk soil for the different soil modalities. The gray lines indicate + 0.4 and 0 (per mile) to guide the eye. Error bars correspond to the 2SD interval (see Statistical Analyses section for details).

Mean Cu isotope ratios in soil solutions include all measured samples with the exception of week 4 (Table 2). Samples of week 4 were excluded because they appeared to show transitional variations in some samples as discussed below. For STM, VI, and late OB samples all replicates were pooled for a given time step. To detect temporal variations, the evolution of one replicate is shown for CI, HBN, and CO. In STM, VI, and CI soil solutions, Cu isotope ratios are heavier than in the corresponding bulk soils (Δ^65^Cu_Solution–Soil_ around + 0.4‰, Figure 6). No Cu isotope fractionation between soil and soil solution was detected in soils from the Bordeaux area (Figure 6). Only in the first analyzed samples (week 4) of HBN and VI, δ^65^Cu values were significantly heavier in soil solution than in bulk soil (Δ^65^Cu_Solution–Soil_ of 0.29 ± 0.09‰ and 0.63 ± 0.03‰, respectively).

### Biomass Production of Grapevine Plants

**FIGURE 7:**
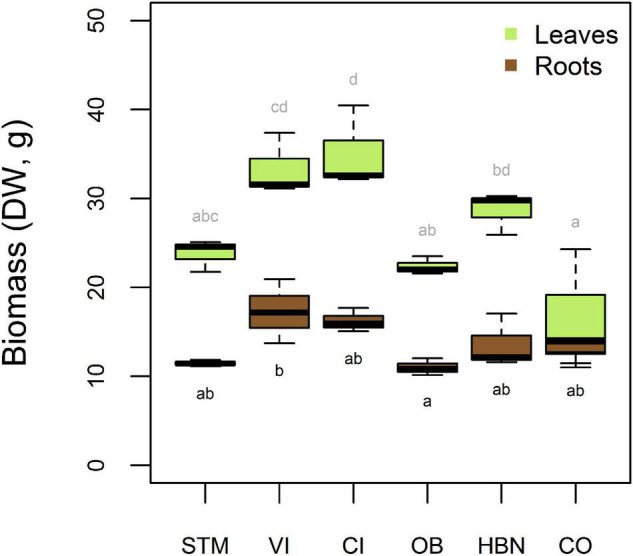
Biomass of roots and leaves of the grapevine plants in dry weight (DW) per soil. Leaf biomasses include young leaves, that were used for Cu analysis, as well as old and senescent leaves that were not analyzed. Significantly different (Tukey’s HSD, α = 0.05) groups are displayed above the boxes for leaf masses and below for root masses.

After 16 weeks of growth, the measured root biomass was between 10.1 and 20.9 g DW (Figure 7). A one-way ANOVA showed a significant influence of soil modality on root biomass (*p* < 0.02). Tukey’s HSD showed that only the mean root biomasses of VI and OB soil were significantly different (*p* < 0.04), with 6.28 g higher root biomass in VI than in OB. For all other samples, differences were not significant (Figure 7).

The leaf biomass was also different according to the soil modality (one-way ANOVA *p* < 0.001). Tukey’s HSD showed that CI samples had a significantly higher leaf biomass than STM (−11.3 g DW, *p* < 0.04), OB (−12.7 g DW, *p* < 0.02), and CO (−18.6 g DW, *p* < 0.001) samples, and VI samples had a higher leaf biomass than OB (−11.0 g DW, *p* < 0.05) and CO (−16.9 g DW, *p* < 0.03) samples. Furthermore, HBN samples had higher leaf biomass than CO (−12.2 g DW, *p* < 0.03) samples. Significantly different groups are shown in compact letter display in Figure 7.

### Copper Uptake From Soil Solution to Roots

**FIGURE 8:**
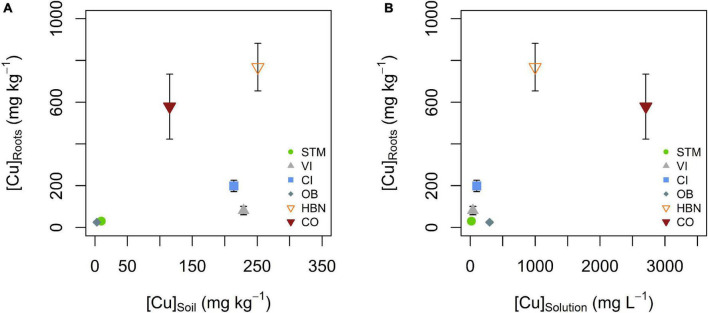
**(A)** Mean root Cu concentration ([Cu]_Roots_) plotted against bulk soil Cu concentration ([Cu]_Soil_). **(B)** Mean root Cu concentration ([Cu]_Roots_) plotted against mean soil solution Cu concentration ([Cu]_Solution_). There was no apparent correlation between [Cu]_Roots_ and [Cu]_Soil_ or [Cu]_Solution_. Error bars correspond to the SD interval around the mean of [Cu]_Roots_ for three replicates per soil modality.

The lowest mean Cu concentrations in roots ([Cu]_Roots_) were detected in STM and OB grown plants with 30 and 25 mg kg^–1^ (Table 2), followed by the Italian soils CI and VI, with [Cu]_Roots_ of 199 and 81 mg kg^–1^. The highest [Cu]_Roots_ were found in CO and HBN soils with, respectively, 579 and 768 mg kg^–1^. The differences in [Cu]_Roots_ were statistically significant between the soil modalities (one-way ANOVA *p* < 10^–9^). Tukey’s HSD showed that with exception of the pairs OB-STM and HBN-CO, [Cu]_Roots_ significantly differed between each soil modality (*p* < 0.002, Table 2). The [Cu]_Roots_ followed the rank of [Cu]_Solution_ (Figure 4) for most soils, only the rank of OB decreased. Except in CI and VI, [Cu]_Roots_ was higher than the [Cu]_Soil_ so that significant contamination by possible leftover soil particles can be excluded. [Cu]_Roots_ was not correlated to [Cu]_Soil_ or [Cu]_Solution_ (Figures 8A,B).

**FIGURE 9:**
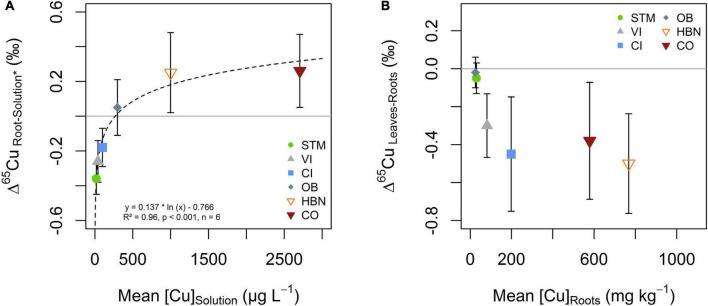
**(A)** Isotope fractionation between soil solution and roots as a function Cu content in the soil solution. Isotope ratios in soil solution excluded the 4 weeks sample (Solution*), due to transient variations discussed (see section “Mobility of Cu Into the Soil Solution and Its Evolution Over Time”). The dashed line represents the fit of the logarithmic function displayed in the figure. **(B)** Cu isotope fractionation between roots and leaves plotted against Cu content in roots. The error bars in both panels correspond to the 2SD interval around the mean of three replicate plants per soil modality.

Copper isotope fractionation between soil solution and roots was increasingly positive with higher [Cu]_Solution_ (Figure 9A). Negative Cu isotope fractionation between roots and soil solutions occurs in STM, VI and CI modalities (Δ^65^Cu_Roots–Solution_ of -0.36 ± 0.09‰, -0.26 ± 0.12‰, and -0.18 ± 0.11‰, respectively). Positive isotope fractionation between roots and soil solution was observed for the Bordeaux soils OB, HBN, and OB (Δ^65^Cu_Roots–Solution_ = 0.05 ± 0.16‰, 0.25 ± 0.23‰ and 0.26 ± 0.21‰, respectively). A logarithmic function (Δ^65^Cu_Roots–Solution_ = 0.137 * ln ([Cu]_Solution_) –0.766) was fitted through all data points (*n* = 6, *R*^2^ = 0.96, *p* < 0.001) (Figure 9A).

### Copper Root-to-Leaf Transfer in Grapevine Plants

Given the large differences of Cu content in soil solutions, between 19 and 2,705 μg L^–1^, the [Cu]_Leaves_ were surprisingly similar in the different modalities, between 4.9 and 10.9 mg kg^–1^. One-way ANOVA showed nevertheless a significant influence of soil modality on [Cu]_Leaves_ (*p* < 0.04). Tukey’s HSD indicated that [Cu]_Leaves_ only differed significantly between STM and CO samples (*p* < 0.04).

When Cu content in roots was low (i.e., OB and STM), no Cu isotope fractionation between roots and leaves occurred (Figure 9B). Modalities with higher Cu content in roots (CI, VI, HBN, and CO) showed light Cu isotope enrichment in leaves compared to roots (Figure 9B).

## Discussion

### Mobility of Cu Into the Soil Solution and Its Evolution Over Time

[Cu]_Soil_ of OB and STM was below average European background concentration of about 14 mg kg^–1^, whereas CO, CI, VI, and HBN soils showed Cu concentrations above the European background level, similar to other Cu contaminated vineyard soils (Chaignon et al., 2003; Lado et al., 2008; Reimann et al., 2018). CI, VI, and HBN exceeded the predicted no-effect concentration (PNEC) for Cu in soils (200 mg kg^–1^) but concentrations were still two to three times less than maximum Cu contents measured in European vineyard soils (Ruyters et al., 2013; Reimann et al., 2018).

Copper mobility in soil solution appeared to be controlled by SOC mobility, rather than by the amount of TOC in the soil solution (Figure 5B). It has long been established that Cu in soil solution is primarily bound to organic matter, however, the role of the mobility of SOC on Cu mobility was less studied (Yin et al., 2002; Chaignon et al., 2003; Ashworth and Alloway, 2004; Ren et al., 2015). In the present study, only soil solutions from the STM soil did not fit into the trend (Figure 5B). This might be linked to the lower pH of STM soil solution, and thus slight differences in Cu speciation in solution or competition between Cu and protons for adsorption sites on dissolved organic matter (Yin et al., 2002; Chaignon et al., 2003; Bravin et al., 2009a,^2012^). STM is also the only soil that did not receive any Cu treatment so that it is possible that only exogenic Cu is controlled by SOM mobility (Wang and Staunton, 2006). This is supported by a correlation between Fe and Cu contents in the STM soil solution (Supplementary Information).

A mobile Cu pool, adsorbed or complexed with organic matter is consistent with a heavy isotopic Cu signature in solution, as observed for STM, CI, and VI (Figure 6; Yin et al., 2002; Chaignon et al., 2003; Ashworth and Alloway, 2004; Vance et al., 2008; Bigalke et al., 2010a; Kusonwiriyawong et al., 2016). The absence of isotope fractionation between soil solution and bulk soils from the Bordeaux area suggests that the total Cu pool in soil and the mobile Cu pool have similar chemical speciation or that desorption does not fractionate Cu isotopes (Figure 6). No isotope fractionation between soil and soil solution would for example be observed if Cu gets into the soil solution by solubilization of the organic molecule to which Cu is bound.

The absence of a [Cu]_Soil_ – [Cu]_Solution_ correlation (Figure 5A) is in contrast with previous studies that found correlations between bulk soil Cu, extractable Cu, or the ionic Cu^2+^ fraction and root Cu (Chaignon et al., 2003; Bravin et al., 2009a,b, 2012). Some studies reported a good correlation between bulk soil Cu and pore water Cu, especially in contaminated soils (Sauvé et al., 2000). However, our observation is perfectly in line with research showing that pH and surface adsorption control Cu mobility, not bulk soil content (Brun et al., 2001; Yin et al., 2002; Chaignon et al., 2003). There was little variation in [Cu]_Solution_ over time compared to water-extractable Cu in soil incubation experiments (Wang and Staunton, 2006). The increase of [Cu]_Solution_ in CI over time might reflect active processes, including rhizosphere acidification, exudation of reducing agents or phytosiderophores, implemented by plants to satisfy needs of Fe nutrition (Kraemer, 2004; Ström et al., 2005). Indeed, CI is the most calcic soil of the experiment and Fe deficiency is common in such soils (Kraemer, 2004; Ström et al., 2005).

In our setting, the offset in isotope fractionation in the first sample is likely due to the rewetting and adaptation of the soil to the new environment, accompanied by a priming effect (Figure 6; Miller et al., 2005). It was formerly reported that the initial DOM released from rewetted soils has a lower affinity to Cu compared to later, more humified DOM (Amery et al., 2007). Detailed interpretation of this effect without further data would be speculative but if present, it suggests that transitory effects on microbiological activity and/or soil organic matter quality can play an important role in Cu mobility and isotope fractionation.

### Copper Uptake and Translocation in Grapevine Plants

#### Biomass Production

The observations that root biomass was not significantly different in the different soils was surprising, as one of the first symptoms of Cu toxicity in plants is reduced and abnormal root growth (Ambrosini et al., 2015). The only significant difference was detected between VI and OB, indicating that inhibition of root growth was strongest at low [Cu]_Roots_. However, OB also showed very high Fe concentrations in the first solution samples, so that reductive conditions and Fe toxicity might have played a role in this modality (Supplementary Information). The modalities CO and HBN with high Cu contents showed lower root biomass than CI and VI samples even though this was not significant at a 95% level (Figure 4). Plants grown on VI and CI soils also had higher leave biomass than most other modalities, suggesting that low and high Cu supplies limited leaf growth.

#### Cu Concentrations in Plant Tissues

Even though soil solution concentrations varied by more than a factor ×100, grapevine leaves had only a narrow Cu concentration range (Table 2). The Cu concentrations reported for the leaves in our study are consistent with the value of around 6 mg kg^–1^ reported in healthy leaves (Marschner and Marschner, 2012). Moreover, despite high Cu content in soil solutions, no signs of toxicity, such as abnormal root morphology, were observed (Ambrosini et al., 2015). Significant differences in [Cu]_Leaves_ were only detected between the least and most concentrated leaves. These observations are in line with former research showing a tight homeostatic control of Cu in plant tissues (Mitchell et al., 1978; Brun et al., 2001; Chaignon et al., 2003; Alaoui-Sossé et al., 2004; Chen et al., 2011; Marschner and Marschner, 2012; Ryan et al., 2013).

#### Relevant Mechanisms of Cu Isotope Fractionation in Plants

To interpret Cu isotope fractionation within plants it is important to consider the equilibrium fractionation of Cu isotopes during reactions that might occur within the plant. At first, the Cu isotope fractionation induced by the complexation of aqueous Cu^2+^ by organic ligands is reported to be heavy. Bigalke et al. (2010a) measured an enrichment of + 0.26 ± 0.11‰ in insolubilized humic acid with respect to solution Cu^2+^. Ryan et al. (2014) reported increasingly heavy isotope fractionation of up to 0.84‰ with increasing complex stability. On the other hand, reactions that included the reduction of Cu(II) to Cu(I) were reported to induce a strong light isotope fractionation of -2.6 to -4 ‰ in the reduced phase (Zhu et al., 2002; Ehrlich et al., 2004; Navarrete et al., 2011). However, one needs to bear in mind that under non-equilibrium conditions, light isotopes tend to react faster than heavy isotopes leading to an enrichment of light isotopes in the reaction products – the so-called kinetic isotope fractionation. This effect can lead to isotope signatures opposite to what would be expected from equilibrium fractionation.

##### Mechanisms of Cu Isotope Fractionation During Root-to-Shoot Transport

Isotope fractionation between roots and leaves was light for samples with high Cu exposure (Figure 9B), consistent with earlier studies showing light Cu isotope enrichment in aerial parts of the plant (Weinstein et al., 2011; Jouvin et al., 2012; Blotevogel et al., 2019). This effect might be due to the retention of heavy Cu in the roots, by vacuole storage or pectin binding as a response to Cu excess. This would lead to the depletion of heavy Cu isotopes in the cytoplasm so that light isotopes would be preferentially transported to the leaves. Another possibility is kinetic fractionation during Cu transport to leaves, which is increasingly negative with increasing Cu availability and transpiration rates (Ryan et al., 2013; Couder et al., 2015; Li et al., 2016). The latter is also in line with observations of very light isotope signatures in leaves of the field grow grapevine plants (Blotevogel et al., 2019).

##### Mechanisms Associated With Light Isotope Uptake

Isotope fractionation at the solution-root interface reveals the dynamic character of Cu uptake by the grapevine roots (Figure 9A). When Cu supply was low, light isotope fractionation occurred at the soil solution-root interface (STM, VI, CI). In earlier studies, light isotope fractionation was associated with active Cu uptake mechanisms including Cu(II) reduction to Cu(I) at the root surface (Jouvin et al., 2012; Ryan et al., 2013). Besides, a study on yeast cells reported a large light isotope fractionation during uptake by wild types when Cu was supplied as Cu(II) (Cadiou et al., 2017). This fractionation was absent when reductase activity was suppressed, but present when Cu(I) was supplied in solution. They concluded that active high-affinity transporters induce a strong light isotope fractionation if sufficient Cu(I) is available. This suggests, that light isotope fractionation during plant uptake is not exclusively due to Cu(II) reduction. The lighter Cu ratios might stem from kinetic fractionation due to the one-way character of high-affinity uptake (Hindshaw et al., 2013). In our setting, kinetic isotope fractionation can likely be ruled out as the light isotope fractionation between soil solution and roots does not increase with increasing Cu supply, so that the fractionation at low Cu exposure is likely due to a combination of Cu(II) reduction and active transport.

##### Mechanisms Associated With Heavy Isotope Uptake

The progressively positive fractionation between roots and soil solutions (Figure 9A) suggests that the part of Cu that is actively taken up decreases with increasing Cu content in soil solution. Preferential uptake of heavy Cu isotopes (OB, HBN, CO) is likely due to the complexation of Cu on pectin groups at the cell wall or storage mechanisms of excess Cu (Ryan et al., 2013). Furthermore, the uptake of complexed Cu from solution or unspecific uptake pathways such as ZIP transporters, YSL transporters, or ion channels could lead to higher isotope ratios in roots than in soil solution (Laurie et al., 1991; Roberts et al., 2004; Ryan et al., 2013; Caldelas and Weiss, 2017). Fitting the logarithmic function displayed in Figure 9A suggests that the [Cu]_Solution_ for which active uptake is lower or equal to unspecific, passive uptake is 270 μg L^–1^. This value was determined as the x-intercept of the function because from this point on negative fractionation from active uptake is completely compensated by positive fractionation from passive uptake. The limit would be reached at even lower concentrations if the fractionation of “pure” active uptake is even more negative than isotope fractionation in STM.

##### Cu Adsorption vs. Cu Uptake

Earlier research suggested that a large part (70%) of root Cu is adsorbed on root surfaces (Ryan et al., 2013; Li et al., 2016). In the apoplast, Cu is mainly bound to carboxyl groups of cell wall polymers or nitrogen groups of cell wall proteins, both favor heavy isotopes (Reilly, 1969; Allan and Jarrell, 1989; Sattelmacher, 2001; Colzi et al., 2012; Ryan et al., 2014; Guigues et al., 2016). The immobilization of heavy Cu appears not to be purely due to adsorption. The absence of correlation between [Cu]_Solution_ and [Cu]_Roots_ shows that [Cu]_Roots_ is already influenced by the plant’s biological response to Cu availability rather than pure physico-chemical factors. For example, pure adsorption of Cu on roots with the same binding sites would result in an adsorption isotherm, that was not observed here. A modification of type and number of pectin molecules at the root surface as a response to Cu exposure or lowered transpiration mass transport might explain this observation (Sattelmacher, 2001; Castaldi et al., 2010; Colzi et al., 2012; Meychik et al., 2016). Further, possible plant responses to Cu exposure include immobilization of Cu *via* metallothioneins in vacuoles or efflux pumping as well as modification of physicochemical properties in the rhizosphere (Zhou et al., 2007; Bravin et al., 2009a,b; Burkhead et al., 2009; Yruela, 2009; Jouvin et al., 2012; Alvarez-Fernandez et al., 2014; Printz et al., 2016).

#### Conceptual Geochemical and Physiological Issues of Isotope Fractionation in Whole Plants Raised by the Data

When the overall plant isotope ratios are different from the soil solution, a heavy or a light part of the soil solution Cu either never got into the plant tissue or have been effluxed. This statement includes the cell wall pores making up the apoplastic pathway. Single organ values always present a convoluted signal of input and out into the organ. We do not present a full mass balance here, because stem Cu contents and isotopic ratios were not measured. However, in our setting we can reasonably well approximate the whole plant δ^65^Cu:

(a) In soils, in which roots were lighter than the soil solution, the leaves had the same isotopic ratios as the roots (STM) or were lighter than the roots (CI, VI). Even though we do not have data on stems, these observations suggest that the whole plants were lighter than the soil solution.
(b) In HBN and CO, in which root Cu was heavier than soil solution Cu, the [Cu]_Roots_ exceed the [Cu]_Leaves_ by more than a factor ×50. Even though leave mass is about ×2–×3 higher than that of roots, only a minor fraction of the plant Cu was transported to the leaves. Therefore, the overall plant value can be approximated by the root δ^65^Cu. This suggests that, in HBN and CO-grown plants, the overall Cu isotope ratio is heavy.

Earlier conceptual models explained only light Cu uptake by the plant, but here heavy enrichment of plants needs also to be considered. With the present data, we cannot clearly identify the responsible mechanisms but some hypotheses can be made.

**Hypothesis 1:** One possibility is that OM complexed Cu from the soil solution – which represents virtually all Cu in our study – might not enter the apoplastic pathway, because even basic units of humic and fulvic acids are larger (10 nm) than cell wall pores (5 nm) and the full molecules are much larger (>500 nm) (Baalousha et al., 2006; Marschner and Marschner, 2012; Klučáková, 2018). This implies that a large part of the complexed heavy Cu is excluded from the apoplastic pathway. The fractionation patterns discussed in section “Relevant Mechanisms of Cu Isotope Fractionation in Plants” therefore might only happen at the root surface, and not in the apoplast. This would be consistent with our data for light isotope enrichment (transporters directly take light Cu from soil solution) and heavy enrichment (root surface is in exchange with soil solution and heavy isotopes are adsorbed). This fractionation pattern is in line with the conceptual model of Jouvin et al. (2012), only adding the constraint of non-penetration of Cu into the root cell wall pores. The apparent contradiction with studies showing large fractions of Cu in the apoplast might be due to the environmental parameters as acid pH or absence of SOM, which allowed for a significant fraction due to ionic Cu^2+^, that was able to penetrate the apoplast in those studies (Ryan et al., 2013; Meychik et al., 2016; Cui et al., 2020).

**Hypothesis 2:** Another possibility for heavy isotope enrichment in plants is efflux pumping of light Cu isotopes. Cu efflux pumping is mentioned in the main reviews of Cu transport in plants, but experimental constraints remain scarce (Sattelmacher, 2001; Printz et al., 2016). Under this hypothesis, a significant fraction of the soil solution Cu could enter the root apoplast with the transpiration flow. Under high Cu exposure, light Cu would be effluxed by specific transporters against the transpiration flow, resulting in heavy Cu accumulation in the plant. This would be in line with the preferential transport of light Cu by specific transporters (Cadiou et al., 2017). Furthermore, the absence of a correlation between [Cu]_Root_ and [Cu]_Solution_ would be explained, because of the active regulation of Cu content by the plant. For light isotope fractionation at low Cu exposure, the fractionation might occur outside of the root, for example through exsudation of reducing agents, that preferentially mobilize light Cu from the solid soil. But light isotope fractionation might also result from the exclusion of very large, strong complexants as described under Hypothesis 1.

## Conclusion

**FIGURE 10:**
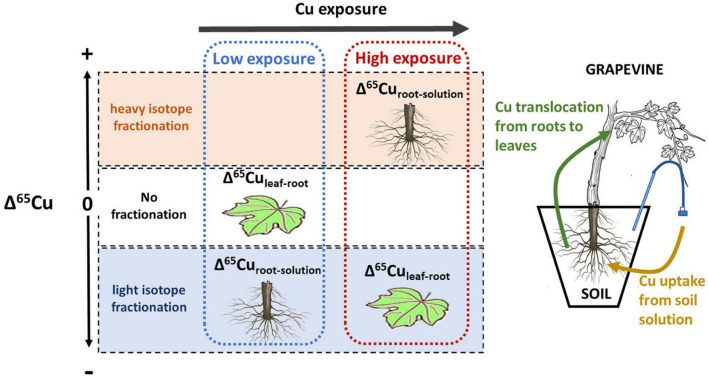
Schematic sketch of Cu isotope fractionation depending on Cu exposure of the plant. At low exposure, Cu isotope ratios in roots are lighter than the soil solution, likely associated with active uptake. No fractionation occurs between roots and leaves. At high exposure root isotope ratios are heavier than the soil solution, likely liked to passive uptake and detoxification. In this scenario leave Cu is lighter than roots.

This study reports results of a 16-week greenhouse experiment growing grapevine plants on 6 soils with different Cu-pesticide treatment histories. The mobility and phytoavailability of Cu in the soil solution were controlled by the solubility of organic matter, not bulk soil Cu content or DOC. Root Cu concentrations showed no direct correlation with bulk soil or soil solution Cu concentration. The Cu-isotope fractionation between soil solution and roots was light for low Cu exposure and increasingly heavy for higher exposure levels, suggesting a progressive change from active to passive uptake (Figure 10). At around 270 μg L^–1^, the isotope fractionation between soil solution and roots changed from light to heavy, indicating that from this value the passive uptake was equal to or higher than the active uptake. Isotope fractionation between leaves and roots was absent for low Cu exposure levels, and light for high exposure.

Our results show that isotope fractionation patterns in roots and leaves are linked to Cu exposure of the plants. In particular, Cu isotope fractionation between roots and soil solution can be used as a specific and sensitive tool to monitor changes in uptake mechanisms.

Besides, isotope ratios in leaves might be used to detect high Cu exposure levels before changes in Cu content occur (Figure 10). This is of particular importance in grapevine as physiological effects on roots are often observed before leaf Cu content increases (Toselli et al., 2009; Ambrosini et al., 2015; Brunetto et al., 2016).

## Data Availability Statement

The original contributions presented in the study are included in the article/Supplementary Material, further inquiries can be directed to the corresponding author.

## Author Contributions

SB, PO, LD, and SA did the soil sampling. SB, PO, LD, and ES participated in and supervised the greenhouse experiments and plant sampling. SB, PO, LD, JV, and ES performed and supervised chemical analysis and sample handling. SB, PO, and ES participated in writing the first draft. PO and ES acquired funding and did the project administration. All authors were involved in reviewing and editing.

## Conflict of Interest

The authors declare that the research was conducted in the absence of any commercial or financial relationships that could be construed as a potential conflict of interest. The reviewer CC declared a past co-authorship with one of the authors JV to the handling editor.

## Publisher’s Note

All claims expressed in this article are solely those of the authors and do not necessarily represent those of their affiliated organizations, or those of the publisher, the editors and the reviewers. Any product that may be evaluated in this article, or claim that may be made by its manufacturer, is not guaranteed or endorsed by the publisher.
