# Supplementary material for: Stable Cu Isotope Ratios Show Changes in Cu Uptake and Transport Mechanisms in Vitis vinifera Due to High Cu Exposure
Source: Front Plant Sci. 2022 Jan 12;12:755944. doi: 10.3389/fpls.2021.755944 (PMC8790286; doi:10.3389/fpls.2021.755944)
Supplement: Supplementary file 1 [file Data_Sheet_1.docx]

**Supplementary Information 1: Details on the Cu purification.**

**Table SI1.** Purification protocols for plant, solution and bulk soil samples. For the purification of plant material and soil solutions the same protocol was applied twice, while the bulk soil procedure had two distinct steps.

|  | **Plant and soil solution samples** |  | **Bulk soil samples** | |
| --- | --- | --- | --- | --- |
|  |  |  | **First step** | **Second step** |
| **Resin Volume** | 2 mL |  | 2.5 mL | 2 mL |
| **Conditioning** | 6 mL 7 M HCl 0.01% H_2_O_2_ |  | 6 mL 7 M HCl 0.01% H_2_O_2_ | 6 mL 7 M HCl 0.01% H_2_O_2_ |
| **Sample loading** | 1 mL 7 M HCl 0.01% H_2_O_2_ |  | 1 mL 7 M HCl 0.01% H_2_O_2_ | 1 mL 7 M HCl 0.01% H_2_O_2_ |
| **Matrix elution** | 9 mL 7 M HCl 0.01% H_2_O_2_ |  | 9 mL 7 M HCl 0.01% H_2_O_2_ | 9 mL 7 M HCl 0.01% H_2_O_2_ |
| **Cu elution** | 24 mL 7 M HCl 0.01% H_2_O_2_ |  | 15 mL 4 M HCl 0.01% H_2_O_2_ | 23 mL 7 M HCl 0.01% H_2_O_2_ |

**Supplementary Information 2: Detailed soil solution analysis.**

In Table SI2, all soil solution measurements of Cu and δ^65^Cu are reported. In addition, for the samples in which concentration measurements were carried out by ICP-MS the Fe concentration is also given. Recovery of Fe in the SLRS5 reference solution was 96 ± 7 µg/L for a certified value of 91.2 ± 5.8 µg/L.

Highest Fe contents were measured in solutions from OB soil, with an decreasing tendency over time (Figure SI1). For VI, HBN and CO there was no significant (p>0.05) correlation between [Fe]_solution_ and [Cu]_solution_ (Figure SI2). For STM there was a good (R²=0.82) linear relation between [Fe]_solution_ and [Cu]_solution_  if the three highest Fe measurements were excluded. In OB [Cu]_solution_  appeared to vary with the log ([Fe]_solution_) (R²=0.75). For CI there was a significant linear correlation between [Fe]_solution_ and [Cu]_solution_, however it relied heavily on the three highest Cu measurements.


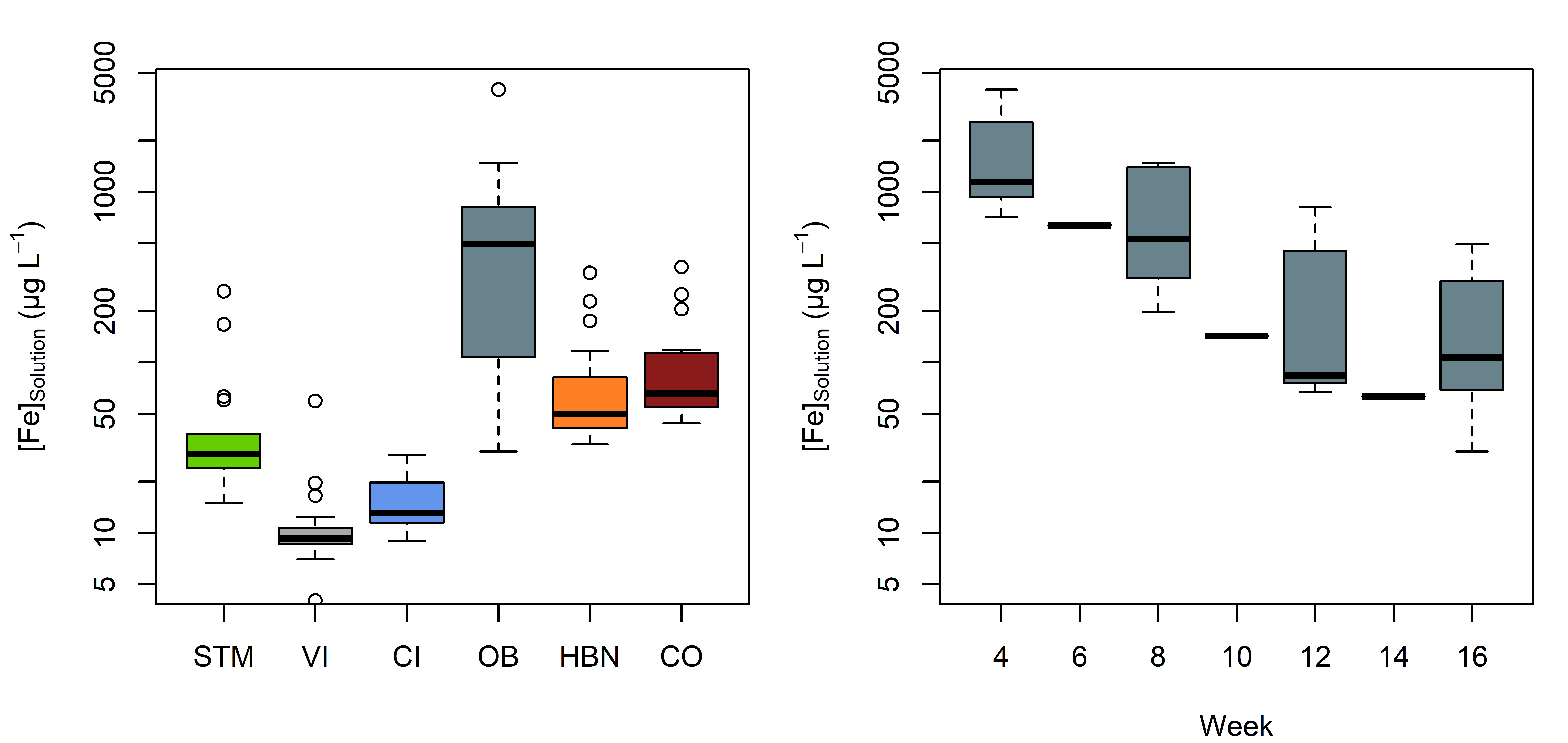


**Figure SI1. (a) Fe concentrations in soil solutions of the different soils. (b) Evolution of Fe in the soil solutions of OB. Horizontal lines indicate time steps with only one measurement.**





**Figure SI2. [Cu]_solution_  of the 6 soil modalities plotted as a function of [Fe]_solution_ . Straight lines indicate best fit regressions. Red points in STM were excluded from the regression. N.S. indicates that the represented regression line was not significant.**

**Table SI2. Soil solution measurements of Cu and δ^65^Cu, by soil and replicate. For the samples in which concentration measurements were carried out by ICP-MS the Fe concentration is also given.**

| **modality** | | **STM** | | | | **VI** | | | | **CI** | | | | **OB** | | | | **HBN** | | | | **CO** | | | |
| --- | --- | --- | --- | --- | --- | --- | --- | --- | --- | --- | --- | --- | --- | --- | --- | --- | --- | --- | --- | --- | --- | --- | --- | --- | --- |
| **week** | **Rep.** | **Cu** | **δ^65^Cu** | **2SD** | **Fe** | **Cu** | **δ^65^Cu** | **2SD** | **Fe** | **Cu** | **δ^65^Cu** | **2SD** | **Fe** | **Cu** | **δ^65^Cu** | **2SD** | **Fe** | **Cu** | **δ^65^Cu** | **2SD** | **Fe** | **Cu** | **δ^65^Cu** | **2SD** | **Fe** |
|  |  | µg/L | ‰ | ‰ | µg/L | µg/L | ‰ | ‰ | µg/L | µg/L | ‰ | ‰ | µg/L | µg/L | ‰ | ‰ | µg/L | µg/L | ‰ | ‰ | µg/L | µg/L | ‰ | ‰ | µg/L |
| 4 | 1 | 20 | 0.52 | 0.11 | 36 | 45 | 0.96 | 0.03 | 9.3 | 87 |  |  | 9.0 | 816 |  |  | 3979 | 887 | 0.12 | 0.10 | 82 | 6012 | 0.28 | 0.10 | 205 |
| 4 | 2 | *dry* |  |  | 63 | 57 |  |  | 12.4 | 86 | 0.63 | 0.12 | 11.4 | 338 |  |  |  | 1488 |  |  | 60 | 1762 |  |  | 362 |
| 4 | 3 | 16 |  |  | 25 | 45 |  |  | 9.7 | 92 | 0.62 | 0.10 | 12.7 | 449 |  |  |  | 1671 |  |  |  | 2246 |  |  |  |
| 4 | 4 | 18 |  |  | 27 | 53 |  |  | 8.6 | 94 |  |  | 9.6 | 281 | 0.2 | 0.04 | 1146 | 1521 | 0.41 | 0.06 | 335 | 5804 | 0.02 | 0.13 |  |
| 4 | 5 | 18 |  |  | 32 | 48 |  |  | 10.7 | 114 |  |  | 20.2 | 253 |  |  | 713 | 917 | 0.30 | 0.17 |  | 4540 | 0.17 | 0.03 | 250 |
| 6 | 1 | *dry* |  |  |  | 34 |  |  | 7.7 | 85 |  |  |  | 695 |  |  |  | 999 |  |  |  | 3463 |  |  |  |
| 6 | 2 | 16 |  |  |  | 58 |  |  |  | 66 |  |  |  | 335 |  |  |  | *dry* |  |  | 65 | 1709 |  |  | 65 |
| 6 | 3 | 11 |  |  | 15 | 32 |  |  |  | 71 |  |  | 19.9 | 437 |  |  |  | 1639 |  |  |  | 1736 |  |  |  |
| 6 | 4 | *dry* |  |  |  | 41 |  |  |  | 69 |  |  |  | 231 |  |  | 636 | *dry* |  |  |  | 3018 |  |  |  |
| 6 | 5 | *dry* |  |  |  | 37 |  |  |  | 97 |  |  |  | 178 |  |  |  | 1162 |  |  |  | 4962 | -0.03 | 0.08 |  |
| 8 | 1 | 22 |  |  | 31 | 39 | 0.59 | 0.12 | 8.8 | 74 |  |  | 15.0 | 807 |  |  | 1481 | 1168 | 0.03 | 0.11 | 48 | 2189 | -0.02 | 0.09 | 75 |
| 8 | 2 | 20 |  |  | 167 | *dry* |  |  |  | 89 |  |  | 13.3 | 436 |  |  | 532 | 873 |  |  | 33 | 1374 | 0.12 | 0.09 | 53 |
| 8 | 3 | 12 |  |  | 25 | 34 |  |  | 16.5 | 74 | 0.63 | 0.07 | 10.9 | 485 |  |  | 1393 | 1206 |  |  | 41 | 1040 |  |  | 109 |
| 8 | 4 | 15 |  |  | 25 | 45 |  |  | 19.6 | 73 |  |  | 9.8 | 232 |  |  | 312 | 1086 | 0.03 | 0.19 | 36 | 2824 | -0.04 | 0.13 | 70 |
| 8 | 5 | 18 |  |  | 60 | 34 |  |  | 9.2 | 104 |  |  | 19.0 | 216 | 0.13 | 0.13 | 197 | 860 | 0.04 | 0.22 | 34 | 3073 | -0.02 | 0.09 | 56 |
| 10 | 1 | 23 |  |  |  | 31 |  |  | 4.0 | 106 |  |  |  | *dry* |  |  |  | 1057 |  |  |  | 2022 |  |  |  |
| 10 | 2 | 15 |  |  |  | 39 |  |  |  | 101 |  |  |  | 344 |  |  |  | 613 |  |  |  | 1346 |  |  |  |
| 10 | 3 | 11 |  |  | 261 | 29 |  |  |  | *dry* |  |  |  | 427 |  |  |  | 949 |  |  |  | 870 |  |  |  |
| 10 | 4 | 12 |  |  |  | 42 |  |  |  | 67 |  |  |  | 152 |  |  | 143 | 1038 | -0.03 | 0.04 | 37 | 3077 |  |  |  |
| 10 | 5 | 21 |  |  |  | 33 |  |  |  | 113 |  |  | 19.6 | 117 |  |  |  | 905 |  |  |  | 3202 | 0.02 | 0.24 | 44 |
| 12 | 1 | 26 | 0.61 | 0.08 | 37 | 28 | 0.53 | 0.10 | 7.0 | 95 |  |  | 12.7 | 536 | 0.25 | 0.11 | 812 | 653 | 0.00 | 0.03 | 79 | 1785 | -0.01 | 0.13 | 66 |
| 12 | 2 | 27 |  |  | 38 | 43 |  |  | 59.3 | 103 |  |  | 13.1 | 245 |  |  |  | 553 | 0.14 | 0.08 | 45 | 1346 | -0.03 | 0.08 | 54 |
| 12 | 3 | 13 |  |  | 24 | 29 |  |  | 10.7 | 76 | 0.49 | 0.11 | 12.7 | 377 |  |  |  | 1395 |  |  |  | 953 |  |  |  |
| 12 | 4 | 14 |  |  | 23 | 42 |  |  | 9.9 | 84 |  |  | 11.5 | 28 |  |  | 67 | 573 | 0.11 | 0.08 | 228 | 2709 | -0.06 | 0.06 |  |
| 12 | 5 | *dry* |  |  |  | 33 |  |  | 8.6 | 147 |  |  | 21.6 | 78 | 0.25 | 0.19 | 84 | 962 | 0.07 | 0.11 |  | 2751 | -0.1 | 0.10 | 118 |
| 14 | 1 | *dry* |  |  |  | 36 |  |  | 8.9 | *dry* |  |  |  | 517 |  |  |  | *dry* |  |  |  | *dry* |  |  |  |
| 14 | 2 | 30 |  |  |  | *dry* |  |  |  | *dry* |  |  |  | 173 |  |  |  | *dry* |  |  |  | *dry* |  |  |  |
| 14 | 3 | 13 |  |  | 24 | 33 |  |  |  | *dry* |  |  |  | 424 |  |  |  | 1439 |  |  | 175 | 1068 |  |  |  |
| 14 | 4 | 24 |  |  |  | *dry* |  |  |  | *dry* |  |  |  | 28 |  |  | 63 | 597 |  |  |  | 2854 |  |  |  |
| 14 | 5 | *dry* |  |  |  | 61 |  |  |  | 170 |  |  | 28.7 | 63 |  |  |  | 892 |  |  |  | 3964 |  |  |  |
| 16 | 1 | *dry* |  |  |  | 31 |  |  | 7.1 | 110 |  |  |  | 215 |  |  |  | *dry* |  |  |  | 6060 |  |  |  |
| 16 | 2 | 28 |  |  |  | *dry* |  |  |  | 135 |  |  |  | 122 |  |  | 107 | *dry* |  |  |  | 1098 |  |  | 57 |
| 16 | 3 | 13 |  |  | 21 | 35 |  |  |  | 159 |  |  |  | 292 |  |  | 493 | 833 |  |  | 48 | 794 |  |  | 52 |
| 16 | 4 | 26 |  |  |  | *dry* |  |  |  | 154 |  |  |  | 21 |  |  | 30 | 469 |  |  | 116 | 2257 | -0.06 | 0.09 | 65 |
| 16 | 5 | *dry* |  |  |  | 43 |  |  |  | 168 | 0.53 | 0.09 | 23.6 | 58 |  |  |  | 715 |  |  | 50 | 5521 | -0.01 | 0.09 |  |
